# Suicidal Ideation and Associated Factors among Adolescents in Northeastern Brazil

**DOI:** 10.1155/2014/450943

**Published:** 2014-11-24

**Authors:** Roberto Jerônimo dos Santos Silva, Fabio Alexandre Lima dos Santos, Nara Michelle Moura Soares, Emerson Pardono

**Affiliations:** ^1^Postgraduate Program in Physical Education, Department of Physical Education, Federal University of Sergipe, Avenue Marechal Rondon S/N, Jardim Rosa Elze, 49000-000 São Cristóvão, SE, Brazil; ^2^Center for Studies and Research, UFS em Movimento, São Cristóvão, SE, Brazil; ^3^Postgraduate Program in Health Sciences, Federal University of Sergipe, Avenue Marechal Rondon S/N, Jardim Rosa Elze, 49000-000 São Cristóvão, SE, Brazil

## Abstract

This study aimed to identify the prevalence and factors associated with suicidal ideation among Brazilian adolescents. The instrument designed for the research was used considering three models with outcomes that identified the following: (a) adolescent had considered suicide, (b) adolescents have planned suicide, and (c) adolescents have attempted suicide. Logistic Regression was used in all models with significance level of 5%. An association between being female and suicidal ideation (OR = 2.18, CI 95% 1.60 to 2.97), suicide planning (OR = 1.80, CI 95% = 1.26–2.56), and suicide attempt (OR = 2.91, CI 95% 1.79 to 4.75) was found. Violent behavior/involvement in fights was associated with thinking about suicide (OR = 2.00, CI 95% = 1.43 to 2.81), suicide planning (OR = 1.65, CI 95% = 1.10–2.46), and suicide attempt (OR = 2.35, CI 95% = 1.49 to 3.70). For cigarette consumption, association was found with suicide ideation (OR = 1.62, CI 95% 1.03 to 2.55), planning (OR = 1.88, CI 95% = 1.15 to 3.08), and attempt (OR = 2.35, CI 95% 1.37 to 4.03). For alcohol consumption, association was found with suicide ideation (OR = 1.93, CI 95% 1.47 to 2.54), planning (OR = 2.22, CI 95% 1.61 to 3.08), and attempt (OR = 1.73, CI 95% 1.15 to 2.59). It was concluded that suicidal ideation was associated with female sex, involvement in fights, and illicit drug use.

## 1. Introduction 

Suicidal ideation is defined as thoughts, ideas, and the desire to commit suicide, being a frequent behavior among adolescents and characterized as a personality disorder or with the character of emotional blackmail [1, 2].

Although suicide rates among children and adolescents in Brazil are relatively low compared with other countries, these rates have increased between 2000 and 2010 and increased from 0.9 to 1.1 per 100 000 children and adolescents, a rate that puts Brazil in the 60th position in a survey conducted in 98 countries on the prevalence of suicides [3].

According to Vidal et al. [4], at some point in life, around 1% to 5% of people may attempt suicide, but the percentage for adolescents can range from 3% to 20% and it has been also emphasized that the risk of suicide increases with the number of attempts. Teixeira and Luis [5] attempted to justify the higher prevalence among adolescents due to the fact that the adolescence is a period of development with biological, psychological, and social changes, accompanied by conflicts and distresses that tend to favor suicidal ideation.

According to Baggio et al. [6], suicidal behavior includes steps such as (a) suicidal ideation; (b) suicide planning; and (c) suicide attempt that may lead to suicide. Of these, the easiest steps to identify at population level, in order to map its prevalence to the elaboration of public policies intended to reduce the suicide rates among adolescents are ideation and attempt itself.

Ideation can be identified from surveys with forms/questionnaires in which anonymity is maintained and which represent the realities that each group of adolescents experiences in their community with aspirations and anxieties. Suicide attempt is only identified in the health care center/hospital and has as limitation the identification of the embarrassment caused to family or close relatives, sometimes leading the act not to be reported.

Thus, ideation is the best feature for mapping in terms of population studies on suicide, because, from the indication of the prevalence and areas where this behavior is presented as more prevalent and/or incident, actions on public health and mental health can be taken in the attempt to avoid the consummation of the act itself. Based on the above, this study aimed to identify the prevalence and factors associated with suicidal ideation among adolescents of Aracaju and metropolitan area (SE).

## 2. Methods

### 2.1. Sampling

This is a cross-sectional epidemiological school-based study with a population of 13373 students enrolled in Public high schools in the state of Sergipe, managed by the State Secretariat of Education and specific Regional Departments of Education of municipalities of Aracaju, Nossa Senhora do Socorro, São Cristóvão, and Barra dos Coqueiros, all in the state of Sergipe, Brazil.

To calculate the sample size, sampling error of 3.0 percentage points, outcome prevalence of 50%, and design effect of 2.0 were adopted. Furthermore, 20% was added for losses and refusals [7]. Based on these procedures, the minimum amount of students investigated to meet the sampling representativeness criteria was 1802.

The sample survey was performed through the cluster sampling in two-stage procedure, considering the administrative distribution according to Regional Departments of Education used by the State Department of Education of the State of Sergipe. In the first stage, high schools of each municipality were considered conglomerates. The number of enrollments should be higher than 350 students, totaling 19 schools across the region observed, with a total of 13373 students.

The second stage was defined by the number of students per classroom conglomerate. The average of 32.62 students per classroom was used; that number was obtained by dividing the total number of students enrolled in eligible teaching units (13373) by the total high school classes in these units (*n* = 410), respecting the proportionality of grades and shifts in each school to draw the number of classrooms per grade.

After stratification, the “*n*” calculated was divided by 32.62 for each unit, reaching the number of classrooms drawn by teaching unit, respecting the proportionality by grades in the teaching unit.

Inclusion criteria were (a) being regularly enrolled in high school of teaching units chosen to participate in the study; (b) having minimum age of 13 and maximum of 18 years; (c) having delivered the informed consent form (ICF) signed by parent or guardian; (d) being present on the day of data collection.

Based on these criteria, the following exclusion criteria were defined: (a) delivering the instrument uncompleted; (b) not having delivered the informed consent form (ICF) signed by parent or guardian; (c) not having completed important data such as age and sex; (d) not having completed at least two-thirds of the instrument used.

### 2.2. Data Collection Procedures

Each teaching unit selected was visited two times. On the first day, the objectives of the study were explained to clarify doubts and to deliver the ICF to students so that parents or guardians could sign it authorizing their participation. Data collection took place on the second day.

For collecting information, the following behaviors were elected: “violent behavior,” “cigarette smoking,” “alcohol consumption,” “marijuana smoking,” “body shape satisfaction,” and “physical activity,” which were identified using an instrument designed for this type of information collection.  Table 1 presents the issues and criteria used for this work.

Socioeconomic variables were gender (male and female), age continuously collected and subsequently dichotomized into ≤16 years and ≥17 years, and socioeconomic status through ABEP questionnaire (2010). The classification of socioeconomic level was grouped into the following strata “High” (“A1,” “A2,” “B1,” and “B2”), “Intermediate” (“C1” and “C2”), and “Low” (“D” and “E”).

### 2.3. Ethical Aspects

This study is complementary to research entitled “*Health risk behaviors among adolescents of Aracaju and metropolitan area*” and was approved by the Ethics Research Committee of the Federal University of Sergipe (CEP/UFS) number CAAE 5724.0.000.107-10.

### 2.4. Data Analysis

For the verification of risk associated with “thinking,” “planning,” and “attempting” suicide, elements of the Lethality Coefficient between “thinking” of suicide and “attempting” the action, between “thinking” and “planning,” and between “planning” and “attempting” suicide were used.

To analyze the association between suicide intent and socioeconomic and behavioral variables, Logistic Regression was performed considering suicide intention as the first model. A second model with intention considered in the suicide planning and a third model with suicide attempt were also adopted. From the crude and adjusted Logistic Regression, odds ratios and confidence intervals of 95% (CI 95%) were estimated. Only variables that in the crude analysis obtained *P* < 0.05 were considered in the adjusted analysis. To be associated with the outcome, significance level of 5% was considered.

## 3. Results

Overall, 2457 questionnaires were applied: (a) 133 (5.41%) were completed by individuals aged 18 years or older; (b) eight (0.33%) were completely blank; (c) 30 (1.22%) were missing important data such as gender and age; (d) 22 (0.9%) had less than 2/3 of answered questions, and 57 (2.32%) did not have parental permission to complete the instrument, totaling 250 (10.18%) who lost or discarded questionnaires. Thus, 2207 (89.82%) of questionnaires applied were eligible for this survey.

Table 2 shows the prevalence of socioeconomic variables and behaviors observed among adolescents of Aracaju and metropolitan area. Among these variables, there is a prevalence of 14% for suicidal ideation, 9.5% for suicide planning, and 5.9% for suicide attempts.

Considering the calculation of the estimated Lethality Coefficient for variable “suicide ideation” and “suicide attempt,” a coefficient of 42.67% was found, indicating concern about the initial behavior of thinking about suicide, because data indicate that two-fifths of adolescents who think about suicide may try this action.

When there is a lethality relationship between “thinking” and “planning” suicide among adolescents, a rate of 67.75% was found, indicating that approximately two-thirds of adolescents who think about suicide can really plan for it. The lethality relationship between “planning” and “attempting” suicide among adolescents was also evaluated, and a rate of 63.28% was found, indicating that two-thirds of adolescents who plan suicide are likely to commit it.

Table 3 shows in the crude model that girls are almost twice more likely to think about suicide than boys, and the same was observed in the adjusted model. When behavioral variables are assessed, it was observed that adolescents with violent behavior are two times more likely to think about suicide, those who consume cigarettes are almost three times more likely to think about suicide, and those who consume marijuana are two and a half times more likely to think about suicide. For the adjusted model, the behavior of thinking about suicide was associated with violent behavior, tobacco use, and alcohol consumption.

For variable “suicide plan” (Table 4), it was verified that females are 50% more likely to plan suicide than males in the crude model, whereas in the adjusted model, this chance is nearly twice. In relation to violent behavior, it was found that “violent” adolescents are almost twice more likely to plan suicide, and this chance falls to almost 50% in relation to those who do not have such behavior. Adolescents who made use of illicit drugs showed higher chances of planning suicide and those dissatisfied with their body shape are one and half times more likely to plan suicide than satisfied ones in both models.

Table 5 shows in the crude model that females are two and a half times more likely to attempt suicide than males, increasing to almost three times in the adjusted model. For the behavioral variables, it was found that violent behavior and illicit drug use increase the likelihood of attempting suicide in both crude and adjusted models.

## 4. Discussion

The main results of this study point to the prevalence of suicidal ideation (thinking and planning) and suicide attempt and high Lethality Coefficient between suicide ideation and attempts and factors that are associated with these behaviors, identifying females, highlighting that adolescents involved in fights and consumers of illicit drugs are more prone to suicidal ideation and attempts, and body shape dissatisfaction was associated with suicide planning.

It was observed that the prevalence of suicidal ideation in this study was lower than that identified in other Brazilian studies [8, 9], showing prevalence of 22.2% in the Northeastern and 36% in the Southern Regions of Brazil.

When considering studies in various parts of the world, a higher prevalence was also found, regardless of the continent observed. In Australian adolescents, prevalence of 16% of suicidal ideation was identified, which increased to 31.3% when considering the thought at some point in the adolescent's life [10]. In Lebanon [11] and North Korea [12] prevalence of 16% and 14.82%, respectively, was found. All studies showed higher prevalence for females in relation to suicidal ideation.

Although the prevalence of suicidal ideation is low when compared to other studies, a high lethality was identified in this survey, pointing out that four out of ten adolescents with suicidal ideation are likely to attempt it. This finding is corroborated by other studies [8, 10, 12, 13], which found that suicidal ideation is strongly associated with the execution of the act, suggesting attention to identify the factors that predispose adolescents to this intention.

As for suicidal ideation, it appears in this study that it is associated with females, violent behavior/involvement in fights, and use of illicit drugs (Tables 2 and 3).

Although there are studies [11, 14] showing association between lower socioeconomic status and older age and suicide ideation and attempt, this study did not identify any association between these variables, which may have occurred because the population of this study has been referenced in Units of the State Public School Network.

Regarding the possible causes of suicidal ideation, studies have indicated that suicidal ideation among adolescents can express distress associated with some internal conflict and indicated as protective factors “to have good affective links,” “the feeling of integration to a group or community,” “religiosity,” “being married or living with a partner,” and “having children” [2, 8, 9, 15]. However, of these protective factors, “good relations,” “social and emotional links,” and “religiosity” seem to be more linked with adolescence [2, 8, 12]. Individual and collective actions aimed at strengthening the protective factors should be performed. The study of Borges and Werlang [9] identified values for ideation among girls checking the same trend of the present work; that is, females tend to be twice more likely to develop this behavior than boys.

In the present study, exposure to alcohol consumption and smoking were associated with suicidal ideation in both sexes. Malta et al. [16] argue that the consumption of alcohol and drug use by adolescents are directly associated with indicators of psychosocial stress such as feelings of loneliness, sleeping problems, feeling of sadness, suicidal thoughts, and suicidal plans, adding that the use of drugs was associated with suicidal plan among boys and suicidal thoughts in both sexes.

In a study conducted with 2552 Australian adolescents in order to identify the prevalence of suicidal ideation and its predictors, it was identified that the use of licit and illicit substances by adolescents is a high risk factor for suicide, since these substances are associated with negative affective states [10].

Studies in various parts of the world [1, 8, 9, 17–19] also point out that, among adolescents, girls are more prone to suicidal ideation and attempts, possibly due to the increased predisposition to depression, reaching a frequency twice higher than boys; however, males were more likely to perform the action related to suicide, experiencing a phenomenon called gender paradox [18]; that is, females have higher prevalence of suicide attempts than males; however, males have better result of the action.

This set of observations points to the need for identification and mapping of the causes that lead adolescents to assume certain behaviors as a way to “escape” from reality. Such identification will lead institutions to implement public policies and actions aimed at this issue.

In the present study, it was identified that females are more likely to attempt suicide than males and violent behavior and illicit drug use tend to encourage this behavior.

Another point to be considered is the fact that women carry out more suicide attempts compared to men because they are more prone to depressive behavior; however, the prevalence of deaths is greater among males because females make use of less violent methods (drugs), which enable greater likelihood of rescue compared to men [10, 12, 20].

Studies [10, 12, 14, 17, 18] indicate that the risk factors for suicide can be due to individual (heredity, biophysiological aspects, mental health, abuse history, history of suicide attempts, and gender), family (family history, some type of family psychopathology, and difficulties in family relationships), environmental and demographic factors (factors associated with social and economic disadvantage, problems at school, etc.), and life stressors (factors that sporadically occur and can enhance any of the listed risk factors) and that some cultural elements should also be considered in attempting to explain this phenomenon.

In the present study, significant association between thought, planning, and attempting suicide and use or consumption of illegal drugs was identified, which led to concern about this behavior among adolescents, suggesting actions at school in this sense.

Another study [13] found that aggressive behavior among adolescents is strongly associated with suicidal ideation, in which adolescents with aggressive behavior are twice more likely to think, plan, and commit suicide than those who do not have this behavior. Several studies have indicated that violent behavior, alcohol consumption, and smoking are directly related to suicide ideation, possibly because adolescents perceive these behaviors as a possible solution to stressful events [6, 9, 12, 13].

In a study that examined alcohol consumption as “light” and “high” and the behaviors of “thinking” and “attempting” suicide, strong association between alcohol consumption and the outcomes observed of “thinking” and “attempting” suicide was identified, even among adolescents who were not classified within a risk group for the involvement of these variables, indicating the risk associated with alcohol consumption and thinking and attempting suicide [21].

The importance of identifying the elements that lead to suicide ideation and attempt is related to possible actions that can be performed in its prevention. In this sense, the study of Avanci et al. [22], considering suicide attempts observed in a hospital in the city of Ribeirão Preto, identified the adolescent who commits suicide as female, aged between 15 and 19 years, white, single with low-skill occupations, and resident of poor neighborhoods, results which are very similar to those found here.

Some studies [8, 22] have reported strong sociocultural influence especially among middle and high-income families in relation to suicide attempt reports, resulting in omissions of attempts or even suicides, which makes preventive and characterization actions difficult.

Although this study has pointed out some paths to follow in the observation, mapping, and intervention on suicidal ideation, the fact that the instrument does not identify why the adolescent thought or planned suicide is a limitation, which could lead to other conclusions or to more effective actions among these young people.

These results point to the need for actions aimed at adolescents, especially females, and greater attention to violent behavior and illicit drug use as a major concern for the prevention of suicide among males.

## Figures and Tables

**Table 1:** Characterization of variables used in this study.

Variables	Question used	Classification
Sex^*^	What is your gender?	Male
		Female

Age^*^	What is your age?	≤16 yeas
		≥17 years

Socioeconomic status^*^	Characterized according to ABEP criteria^†^	High (“A1”, “A2”, “B1” and “B2”)
		Intermediate (“C1” and “C2”)
		Low (“D” and “E”)

Involvement in fights (violent behavior)^**^	During the past 12 months, how often did you get involved in a physical fight?^††^	Never
		One time or more

Alcohol consumption^**^	During the past 30 days, on how many days did you take at least one alcoholic drink?^††^	Never
		One time or more

Cigarette consumption^**^	During the past 30 days, on how many days did you smoke cigarettes?^††^	Never
		One time or more

Marijuana consumption^**^	During your life, how many times have you used marijuana?^††^	Never
		One time or more

Body shape satisfaction^**^	How would you describe your body shape?^††^	Dissatisfied (“very underweight,” “slightly underweight,” “slightly overweight,” “very overweight”)
		Satisfied (“desired weight”)

Physical activity^**^	Characterized from the PAQ-C^‡^ score for the last seven days.	Active (scores above three)

^*^Socioeconomic variables. ^**^Variables related to the behaviors observed; ^†^Economic Classification Criterion Brazil 2010-Brazilian Association of Research Companies; ^††^YRBS-Brazil [23].  ^‡^Physical Activity Questionnaire for Children and Adolescents-PAQ-C [24].

**Table 2:** Sociodemographic characterization and prevalence of risk behaviors among adolescents in Northeastern Brazil, *n* = 2207.

Variable	*n*	%
Gender		
Female	1371	62.1
Male	836	37.9
Age		
≤16 years	1259	57.3
≥17 years	940	42.7
Socioeconomic status		
High (A1, A2, B1, B2)	517	23.7
Intermediate (C1, C2)	1383	63.4
Low (“D” and “E”)	280	12.8
Violent behavior		
Yes	363	16.5
No	1842	83.5
Cigarette smoking		
Yes	146	6.6
No	2051	93.4
Alcohol consumption		
Yes	844	38.5
No	1350	61.5
Marijuana consumption		
Yes	53	2.4
No	2140	97.6
Body shape satisfaction		
Satisfied	1314	59.9
Dissatisfied	879	40.1
Physical activity		
Low levels	1945	89.2
Active	236	10.8
Suicide ideation		
Yes	307	14.0
No	1889	86.0
Suicide plan		
Yes	208	9.5
No	1992	90.5
Suicide attempt		
Yes	131	5.9
No	94.1	94.1

**Table 3:** Crude and adjusted odds ratio for behavior “suicide ideation” among adolescents in Northeastern Brazil, *n* = 2207.

Variable	Crude OR (CI 95%)	*P*	Adjusted OR (CI 95%)	*P*
Gender				
Female	1.85 (1.40–2.44)	<0.01	2.18 (1.60–2.97)	<0.01
Male	1		1	
Age				
≤15 years	0.94 (0.73–1.21)	0.63	—	—
≥16 years	1			
Socioeconomic status				
High (A1, A2, B1, B2)	1.15 (0.74–1.79)	0.60	—	—
Intermediate (C1, C2)	1.14 (0.77–1.69)			
Low (D, E)	1			
Violent behavior				
Yes	2.08 (1.56–2.79)	<0.01	2.00 (1.43–2.81)	<0.01
No	1		1	
Cigarette smoking				
Yes	2.73 (1.84–4.04)	<0.01	1.62 (1.03–2.55)	0.04
No	1		1	
Alcohol consumption				
Yes	2.33 (1.81–2.99)	<0.01	1.93 (1.47–2.54)	<0.01
No	1		1	
Marijuana consumption				
Yes	2.42 (1.29–4.53)	<0.01	1.19 (0.56–2.54)	0.66
No	1		1	
Body shape satisfaction				
Satisfied	1.25 (0.97–1.62)	0.09	—	—
Dissatisfied	1			
Physical activity				
Low levels	1.52 (0.96–2.41)	0.07	—	—
Active	1			

**Table 4:** Crude and adjusted odds ratio for behavior “suicide plan” among adolescents in Northeastern Brazil, *n* = 2207.

Variable	Crude OR (CI 95%)	*P*	Adjusted OR (CI 95%)	*P*
Gender				
Female	1.60 (1.15–2.21)	<0.01	1.80 (1.26–2.56)	<0.01
Male	1		1	
Age				
≤15 years	1.05 (0.78–1.41)	0.76	—	—
≥16 years	1			
Socioeconomic status				
High (A1, A2, B1, B2)	1.24 (0.76–2.03)	0.20	—	—
Intermediate (C1, C2)	0.87 (0.55–1.38)			
Low (D, E)	1			
Violent behavior				
Yes	1.95 (1.38–2.75)	<0.01	1.65 (1.10–2.46)	0.02
No	1		1	
Cigarette smoking				
Yes	3.22 (2.09–4.96)	<0.01	1.88 (1.15–3.08)	0.01
No	1		1	
Alcohol consumption				
Yes	2.72 (2.01–3.68)	<0.01	2.22 (1.61–3.08)	<0.01
No	1		1	
Marijuana consumption				
Yes	2.80 (1.41–5.55)	<0.01	1.19 (0.52–2.75)	0.68
No	1		1	
Body shape satisfaction				
Satisfied	1.45 (1.06–1.99)	0.02	1.40 (1.02–1.93)	0.04
Dissatisfied	1		1	
Physical activity				
Low levels	1.11 (0.68–1.83)	0.67	—	—
Active	1			

**Table 5:** Crude and adjusted odds ratio for behavior “suicide attempt” among adolescents in Northeastern Brazil, *n* = 2207.

Variable	Crude OR (CI 95%)	*P*	Adjusted OR (CI 95%)	*P*
Gender				
Female	2.37 (1.52–3.68)	<0.01	2.91 (1.79–4.75)	<0.01
Male	1		1	
Age				
≤15 years	0.78 (0.54–1.12)	0.18	—	—
≥16 years	1			
Socioeconomic status				
High (A1, A2, B1, B2)	0.94 (0.49–1.80)	0.74	—	—
Intermediate (C1, C2)	1.09 (0.62–1.92)			
Low (D, E)	1			
Violent behavior				
Yes	2.41 (1.61–3.59)	<0.01	2.35 (1.49–3.70)	<0.01
No	1		1	
Cigarette smoking				
Yes	3.78 (2.31–6.17)	<0.01	2.35 (1.37–4.03)	<0.01
No	1		1	
Alcohol consumption				
Yes	2.35 (1.62–3.39)	<0.01	1.73 (1.15–2.59)	0.01
No	1		1	
Marijuana consumption				
Yes	1.38 (0.49–3.88)	0.55	—	—
No	1			
Body shape satisfaction				
Satisfied	1.19 (0.82–1.73)	0.37	—	—
Dissatisfied	1			
Physical activity				
Low levels	1.23 (0.65–2.32)	0.53	—	—
Active	1			

## References

[1] Ahmad N., Cheong S. M., Ibrahim N., Rosman A. (2014). Suicidal ideation among Malaysian adolescents. *Asia-Pacific Journal of Public Health*.

[2] Abasse M. L. F., de Oliveira R. C., Silva T. C., de Souza E. R. (2009). Análise epidemiológica da morbimortalidade por suicídio entre adolescentes em Minas Gerais, Brasil. *Ciência & Saúde Coletiva*.

[3] Mapa da Violência [Internet] http://www.mapadaviolencia.org.br/.

[4] Vidal C. E. L., Gontijo E. C. D. M., Lima L. A. (2013). Tentativas de suicídio: fatores prognósticos e estimativa do excesso de mortalidade. *Cadernos de Saúde Pública*.

[5] Teixeira A. M. F., Luis M. A. V. (1997). Suicídio, lesões e envenenamento em adolescentes: um estudo epidemiológico. *"Revista Latino-Americana de Enfermagem*.

[6] Baggio L., Palazzo L. S., Aerts D. R. G. C. (2009). Planejamento suicida entre adolescentes escolares: prevalência e fatores associados. *Cadernos de Saúde Pública*.

[7] Luiz R., Magnanini M. (2000). The logic of sample size determination in epidemiological research. *Cad Saude Coletiva*.

[8] da Costa Araújo L., Leal Vieira K. F., da Penha de Lima Coutinho M. (2010). Ideação suicida na adolescência: um enfoque psicossociológico no contexto do ensino médio. *Psico-USF*.

[9] Borges V. R., Werlang B. S. G. (2006). Estudo de ideação suicida em adolescentes de 15 a 19 anos. *Estudos de Psicologia*.

[10] Delfabbro P. H., Winefield H. R., Winefield A. H. (2013). Life-time and current suicide-ideation in Australian secondary school students: socio-demographic, health and psychological predictors. *Journal of Affective Disorders*.

[11] Mahfoud Z. R., Afifi R. A., Haddad P. H., DeJong J. (2011). Prevalence and determinants of suicide ideation among Lebanese adolescents: results of the GSHS Lebanon 2005. *Journal of Adolescence*.

[12] Park S. (2013). Gender-specific factors of suicide ideation among adolescents in the republic of korea: a nationally representative population-based study. *Archives of Psychiatric Nursing*.

[13] SouzaI L. D. M., Ores L., de Oliveira G. T. (2010). Ideação suicida na adolescência: prevalência e fatores associados. *Jornal Brasileiro de Psiquiatria*.

[14] Liu R. T., Miller I. (2014). Life events and suicidal ideation and behavior: a systematic review. *Clinical Psychology Review*.

[15] Kamioka H., Tsutani K., Mutoh Y. (2011). A systematic review of nonrandomized controlled trials on the curative effects of aquatic exercise. *International Journal of General Medicine*.

[16] Malta D. C., Souza E. R., de Silva M. M. A. (2010). Vivência de violência entre escolares brasileiros: resultados da Pesquisa Nacional de Saúde do Escolar (PeNSE). *Ciências e Saúde Coletiva*.

[17] Waldvogel J. L., Rueter M., Oberg C. N. (2008). Adolescent suicide: risk factors and prevention strategies. *Current Problems in Pediatric and Adolescent Health Care*.

[18] Langhinrichsen-Rohling J., Friend J., Powell A. (2009). Adolescent suicide, gender, and culture: a rate and risk factor analysis. *Aggression and Violent Behavior*.

[19] Jatobá J. D. V. N., Bastos O. (2007). Depressão e ansiedade em adolescentes de escolas públicas e privadas. *Jornal Brasileiro de Psiquiatria*.

[20] Ficher A. M. F. T., Vansan G. A. (2008). Tentativas de suicídio em jovens: aspectos epidemiológicos dos casos atendidos no setor de urgências psiquiátricas de um hospital ger al universitário entre 1988 e 2004. *Estudos de Psicologia*.

[21] Schilling E. A., Aseltine R. H., Glanovsky J. L., James A., Jacobs D. (2009). Adolescent alcohol use, suicidal ideation, and suicide attempts. *Journal of Adolescent Health*.

[22] Avanci R. C., Pedrão L. J., Júnior M. L. C. (2005). Perfil do adolescente que tenta suicídio em uma unidade de emergência. *Revista Brasileira de Enfermagem*.

[23] Guedes D. P., Lopes C. C. (2010). Validation of the Brazilian version of the 2007 Youth Risk Behavior Survey. *Revista de Saúde Pública*.

[24] Silva R. C. R., Malina R. M. (2000). Nível de atividade física em adolescentes do Município de Niterói, Rio de Janeiro, Brasil. *Cadernos de Saúde Pública*.

